# Influence of action video gaming on spatial representation in the haptic modality

**DOI:** 10.1007/s00221-020-05931-7

**Published:** 2020-09-29

**Authors:** Hanneke I. Van Mier, Hui Jiao

**Affiliations:** Department of Cognitive Neuroscience, Faculty of Psychology and Neuroscience, Maastricht University, Maastricht, The Netherlands

**Keywords:** Action video gaming, Egocentric, Allocentric, Haptic parallelity, Haptic-visual training

## Abstract

Spatial representation in the haptic domain has been shown to be prone to systematic errors. When participants are asked to make two bars haptically parallel, their performance deviates from what would be veridically parallel. This is hypothesized to be caused by the bias of the egocentric reference frame. Stimulating the use of an allocentric reference frame has previously been shown to improve performance in haptic parallelity matching. The aim of the current study was to investigate the influence of action video game experience on parallelity performance. We hypothesized that participants who extensively play action video games with a so-called ‘bird’s-eye view’ are likely to process spatial information more allocentrically, resulting in better performance in haptic parallelity matching. This was tested in two groups of male participants, 10 participants with extensive action video gaming experience (AVGPs) and 10 participants without or hardly any action video gaming experience (NAVGPs). Additionally, the effect of visual–haptic practice on haptic parallelity performance was tested. In the haptic blocks, blindfolded participants had to feel the orientation of a reference bar with their non-dominant hand and had to match this orientation on a test bar with their dominant hand. In subsequent visual–haptic blocks, they had full view of the set-up and visually paralleled both bars. As hypothesized, AVGPs performed significantly better in haptic blocks than NAVGPs. Visual–haptic practice resulted in significantly better performance in subsequent haptic blocks in both groups. These results suggest that playing action video games might enhance haptic spatial representation, although a causative relationship still needs to be established.

## Introduction

Nowadays, an increasing number of people are playing action video games, with quite some players doing this on a weekly or daily basis, playing several hours per week or day. The advent of hand-held gaming devices, tablets, and smart phones makes it possible to play these video games whenever and wherever one wants. Being very experienced in video gaming can even result in becoming a millionaire as was recently evidenced by a 16-year-old winning $3 million playing the video game Fortnite.

The behavioral negative effects of video gaming are often stressed, like addiction and increased aggressive and problem behavior (Gentile et al. 2004; Griffiths et al. 2012; Holtz and Appel 2011), as well as some cognitive negative effects like difficulties sustaining attention (Trisolini et al. 2018) or a reduction in (proactive) cognitive control (Bailey et al. 2010). However, playing action video games has also been shown to enhance performance on various aspects of human behavior and cognition. Positive benefits of action video gaming have been reported for perceptual (Green and Bavelier 2007; Li et al. 2009), motor (Li et al. 2016; Rupp et al. 2019), spatial (Feng et al. 2007; Spence and Feng 2010), as well as cognitive skills, such as executive control (Boot et al. 2008; Strobach et al. 2012), selective attention (Feng et al. 2007; Green and Bavelier, 2003, 2006), working/short-term memory (Colzato et al. 2013; McDermott et al. 2014), and cognitive flexibility (Colzato et al. 2010; Doborwolski et al. 2015). A recent meta-analysis regarding the impact of action video gaming on various domains of cognition reported the three domains with the most robust positive effect as being spatial cognition, top–down attention, and perception (Bediou et al. 2018). Additionally, is has been demonstrated that the benefits of video gaming extended beyond the visual modality. Donohue et al. (2010) found that extensive video game experience led to enhanced multisensory perception, while Green et al. (2010) found improvements in the auditory modality. To our knowledge, the effect of action video gaming in the haptic modality has not been explored. The current study was set up to fill this gap.

Not all studies involving video game players have reported the aforementioned perceptual and cognitive benefits. Murphy and Spencer (2009) were not able to replicate earlier findings that video game playing enhances visual attention. Colzato et al. (2010) reported superior cognitive flexibility in video game players (VGPs) compared to non-video game players (NVGPs) but no improved visual attention. Enhanced working memory was found for VGPs but not inhibition control (Colzata et al. 2013). These inconsistent findings might be due to differences in tasks used to measure cognitive enhancement, different levels of video gaming experience of the participants, or differences in genres of video games played by participants.

A distinction can be made between different genres of action video games, like so-called first-person shooter (FPS) games and real-time strategy (RTS) games, with FPS games being played from a first-person perspective and RTS games being played from a top-down perspective (Doborwolski et al. 2015). The former are thought to support an egocentric perspective, because they are played from the point of view of the player (Spence and Feng 2010), while the latter are assumed to promote an allocentric perspective, because they are played from a so-called bird’s-eye viewpoint (Kühn et al. 2014). It is possible that different game genres impact different cognitive processes and brain areas. The latter is corroborated by structural neural changes related to a change from an egocentric to an allocentric orientation strategy (Kühn et al. 2014). The authors reported volumetric increases in right hippocampus and right dorsolateral prefrontal cortex in a video gaming training group that correlated with the above-mentioned change in orientation strategy. West et al. (2017) also describe a dichotomous effect of video game genre in relation to hippocampal activity, with video games favoring allocentric processing showing increased activation in the hippocampus. Because RTS video games require that multiple targets (stimuli) are tracked simultaneously, often involve fast motion and switching between different screens (viewpoint positions on a map), like FPS games, they qualify as “action video games” as defined by Green and Bavelier (2003) (see Dobrowski et al. 2015). A recent paper (Dale and Green 2017a) described and discussed action game features of more current gaming genres. The authors state that action game characteristics, which were fairly unique to the more traditional action video games of 15–20 years ago, can nowadays also be found in relatively recent genres such as real-time strategy (RTS) games or multi-player online battle arena (MOBA) games with the latter involving action as well as real-time strategy characteristics. In a follow-up study, the same authors reported similar performance on a number of cognitive tasks for action video game players and RTS game players (Dale and Green 2017b). Additionally, Large et al. (2019) showed that experience with MOBA games affected a number of cognitive abilities that are consistent with those reported in existing action video game studies. As the current study will measure the effect of playing RTS video games, we will use the term action video game in the remainder of the paper. However, regardless of the specific video game genre, numerous studies have demonstrated that playing action video games has a beneficial effect on visuospatial cognition (see, e.g., Bediou et al. 2018, for a review). The present study explored the effect of action video gaming on spatial representation in the haptic modality.

While spatial representations in the visual domain has been studied for many decades, interest in studying this in the haptic domain has mainly been shown in the last 2 decades, starting with pioneering studies by Kappers and colleagues (Kappers 1999; Kappers and Koenderink 1999). They studied the haptic perception of spatial relations by asking blindfolded participants to make two bars parallel to each other in the mid-horizontal plane. The participant had to match the orientation of a reference bar, which was fixed and felt with one hand, on a test bar, which had to be rotated with the other hand in the same orientation as the reference bar. The authors reported large and systematic deviations from parallelity. Over the past 20 years, these results have been replicated numerous times in the lab of Kappers (e.g., Kappers 2002, 2003, 2018), in our lab (Kaas and Van Mier 2006; Kaas et al. 2007b; Van Mier 2013, 2016, 2019, 2020) as well as in other labs (Fernandez-Diaz and Travieso 2011; Newport et al. 2002). Although participants report that they feel that both bars are spatially oriented in the same direction when performing this so-called haptic parallelity task, this is almost never the case. Kappers (2003) found deviations ranging over participants from 8° to 91°, with data from our lab showing deviations ranging from 3° to 71° (Van Mier 2013, 2019). The observed deviations are systematic in the sense that they are oriented in the natural direction of the hand, with deviations rotated in a clockwise direction when the test bar is on the right side of the participant (e.g., Kaas and Van Mier 2006; Kaas et al. 2007a; Kappers 2003; Van Mier 2013, 2016, 2019, 2020) and counterclockwise with the test bar being on the left side (e.g., Fernandez-Diaz and Travieso 2011; Kaas et al. 2007b; Kappers 2004, 2018).

Because the deviations in the haptic parallelity task correlate highly with hand orientation, it seems that errors in this task are the result of the biasing influence of the egocentric reference frame. Using an allocentric reference frame in haptic parallelity matching, with space being represented external to the perceiver, would result in bars that would be oriented physically parallel to each other. On the other hand, using an egocentric reference frame, with space represented internal to the perceiver, would lead to bars that would deviate considerably from parallelity. Because the settings in this task have been found to lie in between deviations in an allocentric and egocentric reference frame, Kappers (2003, 2004, 2007) proposed that parallelity in the haptic domain is most likely performed in a frame of reference that is intermediate to an egocentric frame of reference which is mainly centered on the hand, and an allocentric reference frame that is fixed to external space. Furthermore, because the observed deviations are highly participant-dependent, Kappers (2004, 2007; Kappers and Viergever 2006) has argued that haptic parallelity is a weighted average of ego- and allocentric referencing. Participants that depend more on an egocentric reference frame would show larger deviations, while participants that rely more on an allocentric frame would show smaller deviations. This was indeed found, with participants showing larger deviations in the haptic parallelity task having larger egocentric weighting factors than participants with smaller deviations (Kappers 2007). The size of the deviations has also been found to be dependent on the gender of the participant. To date, studies in which gender was included as a factor have reported that the performance of male participants was significantly more veridical than of female participants (Hermens et al. 2006; Kaas and Van Mier 2006; Kappers 2003, 2007; Van Mier 2013, 2016, 2019, 2020; Volcic et al. 2008; Zuidhoek et al. 2007). In addition, job experience has been shown to be related to the size of the deviations (Kappers 2003). Comparing deviations in haptic parallelity matching between male technicians and male physicists or physics students, significant smaller deviations were found for the male technicians. Kappers does not speculate about possible factors that might underlie this difference, but it is possible that technicians are more allocentrically oriented due to their job experience. This brings up the question if male action video game players who play action video games with an allocentric perspective (RTS games with a bird’s-eye viewpoint) perform better in haptic parallelity matching than males without action video game experience, which will be addressed in the current study.

Usually perceptual learning and experience is specific to the trained and performed task with hardly any transfer to other tasks (Barnett and Ceci 2002; Owen et al. 2010). However, transfer effects between action video game playing and perceptual, motor, and cognitive functions and skills have been demonstrated by numerous studies, as mentioned above. It has been demonstrated that learning generalizes beyond the trained activities in these games (Bavelier et al. 2018; Boot et al. 2011; Spence and Feng 2010). It is suggested that playing action video games improves a range of skills that can be applied to other tasks by enhancing general learning capacity (e.g., Bavelier et al. 2012; Green and Bavelier 2012). Additionally, playing those games is thought to enhance attentional resources by increasing awareness to those aspects of the task that are important and decreasing awareness to aspects that are irrelevant (Mishra et al. 2011). Generalization to improved performance in other domains due to action video gaming was shown in a study in which action video gamers were found to be better at lane keeping during driving compared to non-action video gamers (Li et al. 2016). By reviewing studies that looked at a link between action video game usage and surgical skills, Lynch et al. (2010) reported that multiple studies found a positive correlation between playing action video games and improved surgical skill training. The current study was set up to establish if the acquired skills obtained by playing action video games would also show benefits in the haptic modality.

Previous research has shown that it is hard to overcome the biasing influence of the egocentric reference frame in haptic parallelity matching. Participants who had knowledge about and were aware of the deviations in this task performed at the same level as naïve participants (Hermens et al. 2006). Performance hardly improved after visual or haptic training and feedback, and only when combining haptic and visual training, a small improvement was found (Kappers et al. 2008). Recently, Van Mier (2020) looked at the effect of having participants perform the parallelity task visually and haptically in between haptic blocks. Participants started with a regular haptic block, which was followed by a visual–haptic block in which participants had full view of the set-up. In the latter condition, participants were instructed to parallel the test bar to the orientation of the reference bar, and without touching the reference bar, they only looked at it. After this visual–haptic block, they continued with a haptic block, with a total of 11 blocks, six haptic and five visual–haptic blocks, which were presented in alternating order. Van Mier (2020) speculated that participants are making and storing a visual image of the orientations of the bars in the visual–haptic blocks, which they are retrieving and using in subsequent haptic blocks. This visual imagery increases the weight of the allocentric reference frame, resulting in smaller deviations. Although performance in the visual–haptic condition was not veridical, the deviations were much smaller, with a mean around 7.5° for male participants. Being able to see and feel the test hand in the visual–haptic condition significantly reduced the size of the deviations in the haptic condition, but deviations in the latter were still significantly larger than in the former (mean deviation of around 26° for male participants). These results corroborate the rather robust influence of the egocentric reference frame in haptic parallelity matching. It is the question if participants who are less allocentrically oriented would benefit more from visual–haptic practice. This was examined in the current study by comparing the effect of visual–haptic practice on haptic parallelity matching in male participants with and without action video gaming experience. We expected that the former would perform the haptic parallelity task more allocentrically than the latter due to their experience in playing action video games from a top-down allocentric perspective, resulting in smaller deviations. If this would indeed be the case, we hypothesized that participants without action video gaming experience who are less allocentrically oriented would benefit more from visual–haptic practice, showing an overall larger improvement in the haptic condition.

## Materials and methods

### Participants

Twenty male participants with normal or corrected-to-normal vision participated in the study. Half of the participants were classified as habitual action video game players (AVGPs) and the other half as having little or no action video game experience (NAVGPs) based on the Video Game Experience Survey of Terlecki and Newcombe (2005). The groups were matched for age. Mean age of the AVGPs was 23.8 years (SD = 7.4), ranging from 19 to 44 years, while the mean age of the NAVGPs was 25.1 years (SD = 11.3), ranging from 19 to 55 years. Handedness was assessed by a Dutch translation of Annett’s (2004) hand preference questionnaire. Two AVGPs and one NAVGP were left handed, and the other participants showed right-handed dominance. Participants were recruited among students at Maastricht University and (gaming) friends of the second author and received course credits or a monetary reward. Written informed consent was obtained from all participants prior to the experiment. Approval for the study had been obtained by the local ethics committee and the study was performed in line with the principles of the 1964 Declaration of Helsinki.

The criterion to be included in the AVGP group was having played action video games for at least 5 years with a minimum of 5 h per week and still playing at the time of testing. Nine AVGPs had played action video games for more than 10 years. All AVGPs had played a minimum of 10 h per week in the previous years. At the time of testing, three AVGPs played less than 5 h a week (1.5–2 h), but had done so for only a period of around 6 months, while playing 15–25 h per week before that period. All participants in the AVGP group played real-time strategy (RTS) games with action game elements as described in the introduction, while some played also first-person shooter (FPS) games and one participant played mainly FPS games and occasionally RTS games. NAVGPs played no action video games at all (7 participants) or only 0.5–2 h per week for a short period, mainly playing FPS games. At the time of testing, participants in the AVGP group played on average 9.5 h (SD = 8.6) per week, while participants in the NAVGP group played 0.3 h (SD = 0.6) per week. This difference was significant [*t* (18) = − 3.38, *p* = 0.003]. We would like to add that we selected the participants in the NAVGP group based on not extensively playing or having played RTS games or another genre that could be described as an action video game.

Potentially interested participants were verbally questioned about their action video game experience before being included in the study. If they seemed to fulfill the above-mentioned criteria, they were asked to fill in the hereafter mentioned Video Game Experience Survey. All 20 participants who filled in this survey were included in the study.

### Materials and set-up

Video game experience was assessed by the Video Game Experience Survey. This survey is part of the Survey of Spatial Representation and Activities (SSRA) of Terlecki and Newcombe (2005) and is available through the National Science Foundation Spatial Intelligence and Learning Center (https://www.silccenter.org). Analyses have shown that the SSRA is an effective measure of video game playing and experience (Terlecki and Newcombe 2005; Terlecki et al. 2008). The original survey consists of 14 questions assessing video gaming and experience. We skipped questions 11, 13, and 14 who were related to marketing aspects of video gaming. We included an additional question asking how many hours participants play per week on average, resulting in 12 questions in total. One of the questions asked to name the genres of video games that participants play which could be chosen from a given list of video game genres or participants could add their own. Additionally, they had to name the games they were playing. We were mainly interested in how long a participant had been playing video games, how often they played and how many hours per week, and which video games and genres they played. The level of action video game expertise was self-reported. All participants in the AVGP group reported that they were very good or good in playing these games.

**Fig. 1:**
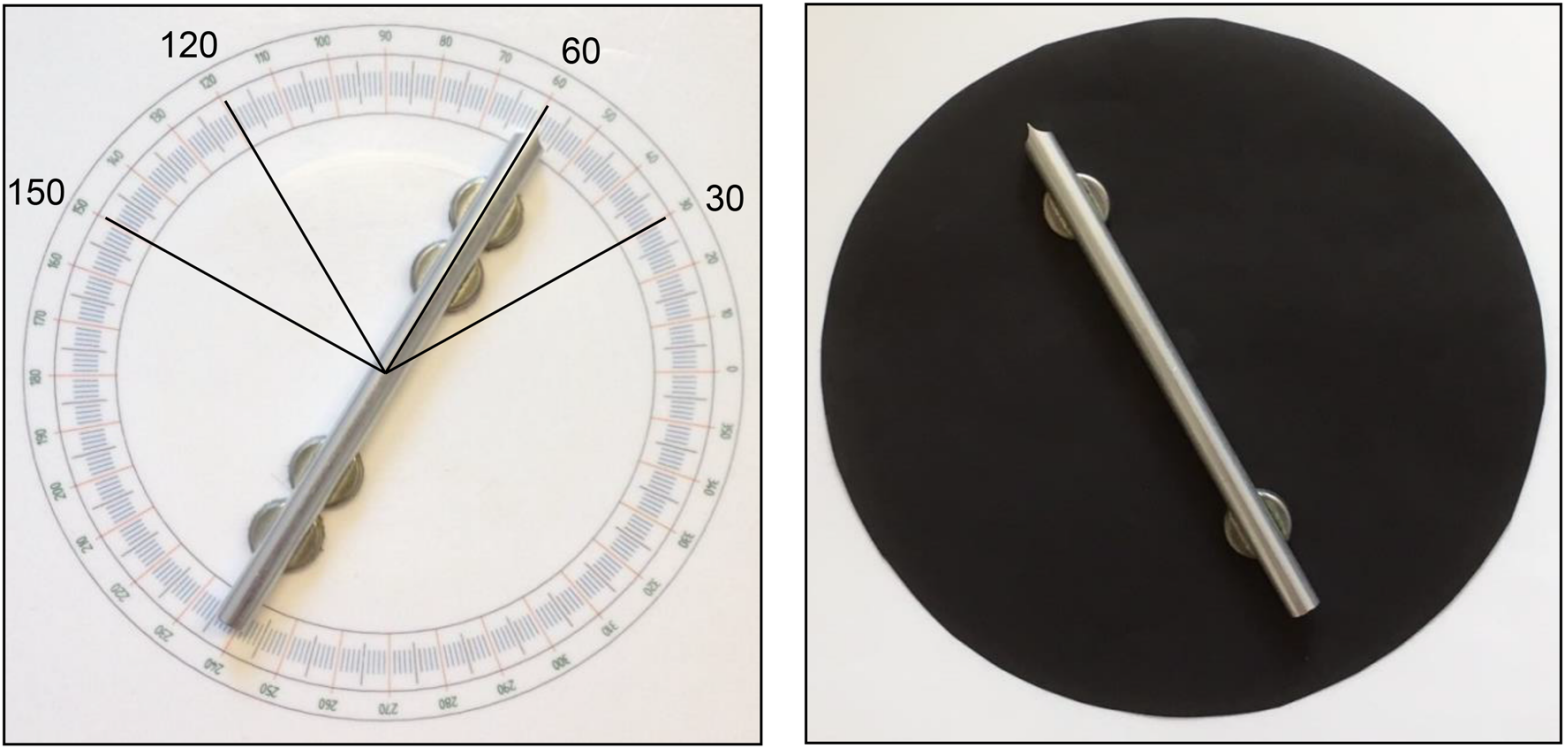
The protractor with the four orientations used in the study. The reference bar with the four magnets is shown in the left picture and the test bar with the two magnets in the right picture. The latter shows a covered protractor. Participants always saw the covered protractors in the visual–haptic condition

For the parallelity task, the same set-up and apparatus was used as in our previous study (Van Mier 2020) and consisted of two square boards of 30 × 30 cm that were covered with a plastic layer on which a protractor with a radius of 10 cm was printed (see Fig. 1, left side). An aluminum bar with a length of 20 cm and a diameter of 1.1 cm was placed on each board. The bar could be easily rotated by means of a small pin attached to the bar that fitted into a small hole in the center of the protractor. Small magnets were attached to the underside of the bars, which increased the resistance to accidental movements due to a thin iron plate that was placed between the board and the plastic layer. The reference bar had two extra magnets to avoid unintentional rotation of this bar. At one end of each bar, an arrow-shaped point enabled accurate setting of the orientation of the reference bar as well as accurate reading of the orientation of the test bar of about 0.5°. Anti-slip mats were placed under the boards to avoid displacement of the boards during the experiment.

The boards were placed on a table in front of the participant at equal distance from the midline of the body of the participant. The distance between the centers of the boards was approximately twice the arm length of the participant. In the visual–haptic condition, the protractors were covered by a circular sheet of black paper (see Fig. 1, right side). This was done to avoid that participants could see the protractor and would match the orientations solely based on the information from the protractor. The black paper covered the protractor precisely, so the experimenter could easily lift the edge of the paper and check the orientation that had to be set or had to be read without the participants seeing the protractor under the black paper.

### Experimental conditions

In the current study, a haptic condition was used, consisting of six blocks, and a visual–haptic condition, which consisted of five blocks, resulting in a total of 11 blocks. In the latter condition, participants received visual as well as haptic feedback while feeling and rotating the test bar. Participants started with a haptic block, which was followed by a visual–haptic block, with both blocks alternating thereafter, and ended with a haptic block. Participants were blindfolded during each haptic block in which they had to match the orientation of the bar on the reference board which was felt with the non-dominant hand, with the orientation of the bar on the test board which had to be rotated with the dominant hand. They were instructed to place both hands simultaneously on the bars. During each visual–haptic block, they had full view of the set-up and the instruction was to look at the orientation of the reference bar without feeling this bar and to match this orientation on the test bar using the dominant hand. Therefore, in the visual–haptic condition, participants could both see and feel the orientation of their (test) hand. In both conditions, participants were instructed to place their hand on the bar with the middle finger resting along the bar to make sure that differences between both conditions could not be related to differences in hand position at the test bar.

Four different reference orientations were used: 30°, 60°, 120°, and 150° (see Fig. 1) to present variation in stimulus presentation. To prevent that participants would recognize cardinal orientations of 0° and 90° in the visual–haptic blocks and use the sides of the plates and/or table to align the test bar to, or use this information in the haptic blocks, these orientations were not used. Because participants were informed that the same orientations would be used in all blocks, we wanted to avoid that they would rather set the test bar in a vertical orientation or horizontal orientation in the haptic condition instead of trying to parallel these orientations after feeling the orientation on the reference bar. The cardinal orientations were used as starting positions for the test bar with the side of the bar with the arrow being directed either to the right or upwards and all orientations being presented with each of the two starting positions. The order and repetition of the orientations was randomized within each block and for each participant, ensuring that the same orientation was never presented consecutively. The experiment took about 1.5 h with each block consisting of eight trials with two repetitions of each orientation, resulting in a total of 88 trials. Half way through the experiment, a break was included.

### Procedure

Participants received general information about the study, and when they agreed to participate in the study, they were asked to fill in and sign the consent form. To demonstrate their understanding of parallelity, they were asked to line two pens in such a way that they were parallel to each other using different orientations. Next, the length of the participant’s arm was measured from the top of the shoulder to the wrist. The centers of both boards were positioned at a distance of approximately twice the arm length, which was determined as described in Van Mier (2020). The average distance between the centers of the boards was 91 cm for AVGPs and 92 cm for NAVGPs. This difference was not significant [*t*(18) = 0.58, *p* = 0.57]. After the third haptic block, between block 5 and 6, a short break of about 10 min was included. Participants started with a haptic block during which they were blindfolded. During the first trials, the experimenter positioned the hands of the participant just above the bars. After each haptic block, participants took off the blindfold to have full view of the set-up during the visual–haptic blocks. Although the protractors were covered during the visual–haptic blocks (see Fig. 1, right picture), participants had to close their eyes between trials when the experimenter had to change the orientation of the reference bar for the next trial. Participants did not receive feedback on their performance.

### Statistical analysis

The dependent variable in all analyses was the deviation between the orientation of the reference bar, which was felt with or located at the side of the non-dominant hand and the orientation of the test bar, which was rotated with the dominant hand. Deviations counterclockwise to the reference bar were noted as negative values, with positive values being noted for clockwise deviations. For the left-handed participants, clockwise deviations were noted as negative and counterclockwise as positive. We averaged over the two repetitions and four orientations. A first repeated measurement ANOVA was performed with Condition (2: haptic vs visual–haptic) and Practice (2: the first vs last block of each condition) as independent within factors and Group (2; AVGP vs NAVGP) as independent between factor. Because the number of blocks for the haptic and visual–haptic condition was not the same, we performed separate analyses for each condition with Practice (6 or 5 blocks) as within variable and Group as between variable. Partial eta-squared (*η*_*p*_^2^) was used to calculate effect sizes for condition and practice, Cohen’s *d* to measure effect sizes for group. Because of the rather small sample size, we assessed normality for each group using the Shapiro–Wilk test. The results of this test showed that deviations were normally distributed in both groups in the haptic condition and all haptic blocks and for NAVGPs in the visual–haptic condition and all visual blocks. Normality was not obtained in the visual condition for AVGPs (*p* = 0.46). This was due to one participant in the AVGP group who had deviations close to zero in blocks 1, 4, and 5 in the visual–haptic condition. Levene’s tests showed homogeneity of error variances for both groups for all six haptic blocks, and for three visual–haptic blocks. Homogeneity was not obtained for blocks 2 and 3 in the visual–haptic condition. This was due to a participant in the NAVGP group, showing much lower deviations in those blocks than the other participants. When sphericity was violated, Greenhouse–Geisser correction was applied, while Bonferroni-corrected post hoc comparisons were used to identify main effects.

## Results

### Effect of video gaming and practice

**Fig. 2:**
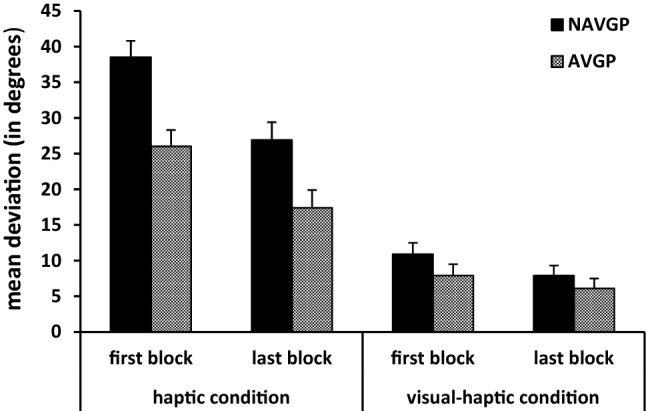
Mean deviations and standard errors for the first and last block in the haptic and visual–haptic condition for AVGPs and NAVGPs

To measure the effect of video game experience on practice in the haptic and visual–haptic condition, an analysis was done including the first and last block of each condition. A significant main effect of group was found [*F*(1,18) = 10.69, *p* = 0.004] as well as a significant two-way interaction of group × condition [*F*(1,18) = 6.64, *p* = 0.019]. AVGPs had a mean deviation of 21.7° and 7.0° in the haptic and visual–haptic condition, respectively, while NAVGPs had mean deviations of 32.7° and 9.4°. Effect sizes (Cohen’s *d*) for group were 0.90 for the haptic condition and 0.06 for the visual–haptic condition. The main effect of condition was significant [*F*(1,18) = 128.34, *p* < 0.001, *η*_*p*_^2^ = 0.88] with mean deviations of 27.2° for the haptic condition and 8.2° for the visual–haptic condition. A significant main effect of practice was also found [*F*(1,18) = 24.64, *p* < 0.001, *η*_*p*_^2^ = 0.58], showing a mean deviation of 20.8° in the first block and 14.6° in the last block. The two-way interaction of group × practice and the three-way interaction of group × condition × practice were not significant (*p* = 0.416 and 0.677, respectively), showing that participants with and without action video gaming experience had similar practice effects in both conditions, as shown in Fig. 2. The interaction between condition × practice was significant [*F*(1,18) = 11.24, *p* = 0.004], with practice effects being more pronounced in the haptic condition than in the visual–haptic condition.

### Effect of video gaming and practice in the haptic condition

**Fig. 3:**
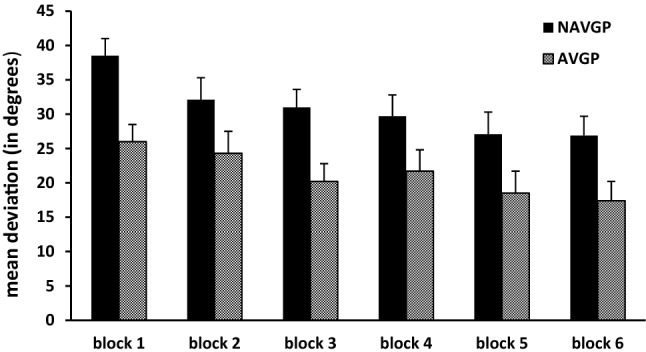
Mean deviation and standard error for the six blocks in the haptic condition for NAVGPs and AVGPs

Because we found a significant interaction of condition × group, we performed two separate analyses per condition. In the analysis for the haptic condition, all six blocks were included. A significant main effect of group was found [*F*(1,18) = 7.17, *p* = 0.015]. Participants in the NAVGP group had a mean deviation of 30.9° when haptically matching the orientations, while participants in the AVGP group had a mean deviation of 21.3°. The effect size (Cohen’s *d*) of group was 0.80. For comparison reasons, we report the mean deviations of the first block, being 26.0° for AVGPs and 38.5° for NAVGPs. Regarding the effect of practice, Mauchly’s test of sphericity indicated that the assumption of sphericity was violated, we therefore used the Greenhouse–Geisser correction. The main effect of practice was significant [*F*(2.7, 47.8) = 10.85, *p* < 0.001, *η*_*p*_^2^ = 0.38], with mean deviations of 32.2° for the first block, 28.2° for the second, 25.6° for the third, 25.7° for the fourth, 22.8° for the fifth, and 22.2° for the last block. Bonferroni-corrected pairwise comparisons between the blocks showed that deviations in the first block were significantly larger than in blocks 3, 5, and 6 (*p* < 0.001). The two-way interaction of group × practice was not significant [*F*(2.7, 47.8) = 0.67, *p* = 0.559, *η*_*p*_^2^ = 0.04]. In both groups, the same pattern of improvement was observed due to having performed the task visually and haptically between the haptic blocks (see Fig. 3).

### Effect of video gaming and practice in the visual–haptic condition

**Fig. 4:**
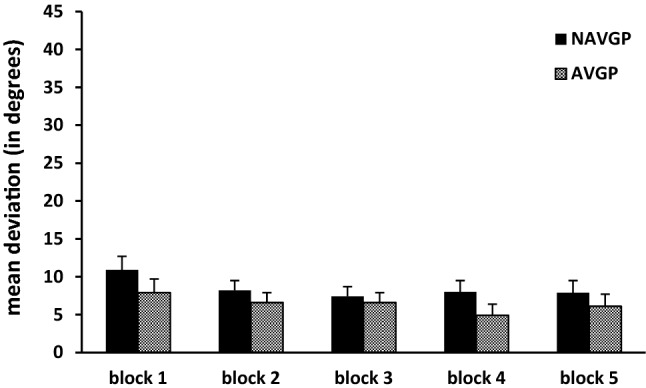
Mean deviation and standard error for the five blocks in the visual–haptic condition for NAVGPs and AVGPs. For this figure, the same scale was used as in Fig. 3 to stress the difference in deviations between both conditions

Because of the significant interaction of condition × group, we also performed a separate analysis for the visual–haptic condition to study the effect of video game experience and practice in this condition. In the analysis for the visual–haptic condition, all five blocks were included. The main effect of group was not significant [*F*(1,18) = 1.22, *p* = 0.285], with NAVGPs showing a mean deviation of 8.5° when visually and haptically matching the orientations, while AVGPs had a deviation of 6.4°. The effect size (Cohen’s *d*) for group was 0.04. With respect to the effect of practice, Mauchly’s test of sphericity indicated that the assumption of sphericity was violated, so we used the Greenhouse–Geisser correction. The main effect of practice was significant, although this effect was much smaller than in the haptic condition as evidenced by the lower effect size [*F*(1.6,29.7) = 3.83, *p* = 0.041, *η*_*p*_^2^ = 0.18]. A deviation of 9.4° was found for the first block, 7.4° for the second, 7.0° for the third, 6.5° for the fourth, and 7.0° for the last block. Bonferroni-corrected pairwise comparisons between the blocks showed that none of the comparisons was significant. Additionally, both groups showed a similar pattern of improvement over the visual–haptic blocks (see Fig. 4) as shown by the non-significant two-way interaction of practice × group [*F*(1.6,29.7) = 0.66, *p* = 0.497, *η*_*p*_^2^ = 0.04]. We additionally tested if deviations in the last visual–haptic block were still significantly different from zero, which was indeed found [*F*(1,18) = 37.77, *p* < 0.001, *η*_*p*_^2^ = 0.68]. The interaction with group was not significant, showing that this was the case for both groups. The same scale was used in Figs. 3 and 4 to stress the difference in deviations between both conditions.

**Fig. 5:**
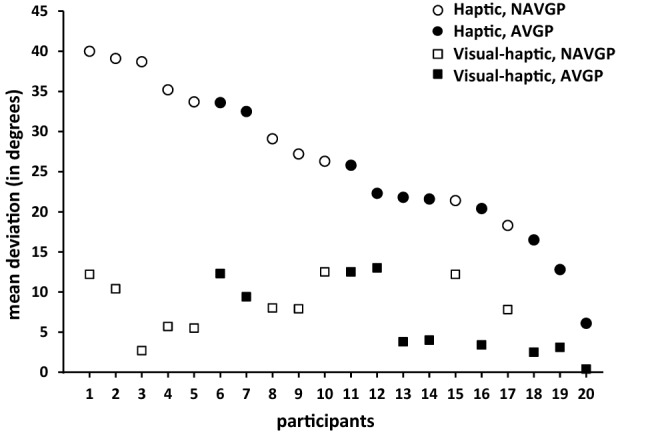
Mean deviations for all 20 participants obtained in the haptic and visual–haptic condition, ordered by the size of the deviations in the haptic condition

The mean deviation over the six haptic blocks and five visual–haptic blocks for each participant is shown in Fig. 5. Deviations were averaged over 48 trials in the haptic condition and 40 trials in the visual–haptic condition. Deviations in the haptic condition ranged from 40.0° to 6.1°, and that in the visual–haptic condition from 13.0° to 0.6°. This figure clearly shows that the largest deviations in the haptic condition were observed for NAVGPs, while deviations in the visual–haptic condition were more similar for both groups.

## Discussion

The current study suggests that enhanced spatial processing due to playing action-based video games might also transfer to improved spatial representation in the haptic domain. Numerous studies have shown a strong link between action video game play and enhanced spatial representation in the visual domain (e.g., Bediou et al. 2018; Spence and Feng 2010). To our knowledge, the effect of action video gaming has not been studied in the haptic modality. We filled this gap by asking blindfolded participants to orient a test bar in such a way that it would be parallel to a set orientation on a reference bar. We hypothesized that AVGPs, who play action video games with a so-called bird’s-eye view, might be more allocentrically oriented which would benefit their performance in the haptic parallelity task. We found indeed that experienced action video game players (AVGP) outperformed non-players (NAVGP) in the above-mentioned haptic parallelity task. Deviations from veridicality were significantly lower in the former group. The high effect sizes of group for the haptic condition suggest that this is a reliable effect. An alternative explanation for the observed results is the possibility of a population bias, being that people who have better spatial processing spend more time playing action-based video games. Although a causal link between playing action video games and enhanced spatial processing has been established for the visual modality, making it likely that this link will also exist in the haptic domain, we would like to stress that causality for the latter still needs to be established.

Performance in the haptic parallelity task has been shown to be biased by the egocentric reference frame (e.g., Kappers 2003, 2004, 2007; Van Mier 2014). The weighting of the influence of this reference frame seems to be participant-dependent (Kappers 2007). Although AVGPs had significantly smaller deviations than NAVGPs, their performance was not veridical, indicating that their performance was still biased by egocentric referencing, although to a smaller extent than NAVGPs. AVGPs had a mean deviation of 26° in the first haptic block compared to 38.5° for NAVGPs. These deviations are close to the reported deviations for (male) technicians (28.2°) and (male) physicists (41.9°) in Kappers’ (2003) study. Although we cannot rule out that this difference in performance in Kappers’ study might be due to the tested technicians being action video game players compared to the physicists not playing such games, it is reasonable to assume that the former were more allocentrically oriented due to their job requirements.

The benefit of having extensively played action video games to haptic spatial processing seems to persist, considering the fact that three of the AVGPs in the current study had been playing only 1.5–2 h per week at the time of testing for a period of around 6 months, but had played 15–25 h per week in the years before. These AVGPs had low mean deviations in the haptic condition (being participants 14, 16, and 20 in Fig. 5). On the other hand, the NAVGP who played only 2 h per week had much larger haptic deviations (participant 5 in Fig. 5). This finding is in line with results reported by Feng et al. (2007) and Li et al. (2009) who found similar transfer effects due to playing action video games after an interval of about 5 months or longer of no gaming.

Considering that action video gaming has been shown to improve visual spatial processing (Green and Bavelier 2006, 2007; Quaiser-Pohl et al. 2006; Spence and Feng 2010), one might have expected also significantly smaller deviations for AVGPs compared to NAVGPs in the visual–haptic condition. Although AVGPs had somewhat smaller deviations compared to NAVGPs, this difference was not significant. This might be due to the small sample size; however, this non-significant finding is in line with results from a previous study in which we compared deviations in a haptic and visual–haptic condition in male and female participants (Van Mier 2020). While males had significantly smaller deviations than females in the haptic condition, their performance was not significantly different in the visual–haptic condition. In the current study, no enhanced performance was found for AVGPs in the visual–haptic blocks. This might be caused by the fact that performance was almost optimal in this condition showing a floor effect in both groups. It is known that also visual space is not veridical (Cuijpers et al. 2000, 2003), so the observed deviations might be due to distortions in visual space. While the main effect of practice in the visual–haptic condition was significant, comparisons between the blocks were not significant, suggesting that there was not much improvement between blocks. Additionally, because this condition was performed visually and haptically, both groups could use external allocentric cues from the environment, like the sides of the plates and table, wall, doors etc. benefitting both groups to the same extent. This might be comparable to findings of studies in which equal performance was found for video game players and non-video game players in conditions where exogenous cues were used (Castel et al. 2005; Dye et al. 2009; Hubert-Wallander et al. 2011). Better performance paralleling both bars visually, however, resulted in enhanced haptic performance only in participants in the AVGP group, as mentioned above.

Manipulations favoring more allocentric processing (Newport et al. 2002; Van Mier 2013; Volcic et al. 2008; Zuidhoek et al. 2003, 2004, 2007) or decreasing the influence of the egocentric reference frame (Kappers and Schakel 2011; Van Mier 2013, 2016, 2019) have resulted in improved parallelity performance. To stimulate allocentric referencing in subsequent haptic blocks in the current study, we included a visual–haptic block after each haptic block. Due to the fact the participants could see and feel the orientation of the test bar, performance in haptic blocks improved, as has also been reported by Van Mier (2020). However, this effect was the same in AVGPs and NAVGPs. We expected that NAVGPs would benefit more in the haptic blocks due to the combined visual and haptic practice in the visual–haptic blocks than AVGPs, but we found that both groups improved at more or less the same rate. This was also observed in the visual–haptic blocks. Although performance improvements were much smaller in the visual–haptic condition, they showed a similar pattern in both groups. We expected that haptic performance in NAVGPs would show larger improvements than in AVGPs due to the former benefitting more from a shift to more allocentric referencing due to visual imagery after having performed the task visually and haptically. Seemingly, NAVGPs and AVGPs use the visual and haptic information obtained in the visual–haptic blocks to the same extent to employ a more allocentric reference frame in upcoming haptic blocks. It has been suggested that due to playing action video games, AVGPs might have an enhanced learning ability due to improved probabilistic or statistical inference (Bavelier et al. 2012, 2018; Green and Bavelier 2012) or enhanced attentional resources focusing on relevant information and ignoring irrelevant information (Green and Bavelier 2012, 2015; Mishra et al. 2011). It may be that AVGPs in the current study showed the same practice-related improvement as NAVGPs due to their enhanced learning and/or attentional capacity. Because of improved selective attention, AVGPs might be better able to attend to the more task-relevant allocentric reference frame while suppressing the irrelevant and biasing influence of the egocentric reference frame than NAVGPs.

Caution is needed interpreting the results of the current study due to the small sample size; however, we would like to stress the high effect sizes of group in the haptic condition (being 0.90 when including the first and last haptic block and 0.80 when including all haptic blocks) and low effect sizes in the visual–haptic condition (being 0.06 when including the first and last visual–haptic block and 0.04 when including all visual–haptic blocks). Although all AVGPs indicated that they were good or very good at playing the action video games, their level of expertise was self-reported. We would also like to stress that we were unable to directly investigate the causal effect of playing action video games on spatial relations in the haptic domain, because we did not measure performance in haptic parallelity matching in NAVGPs after they were trained on RTS action video games. While a causal relationship between playing action video games and enhanced perceptual and cognitive abilities in the visual domain has been established by training/intervention studies (e.g., Feng et al. 2007; Green and Bavelier 2003, 2006; Green et al. 2012; Spence et al. 2009; Strobach et al. 2012), as far as we know, no (training) studies have been performed in the haptic domain. A future direction is to include more participants, to take into account a measurement of gaming expertise and to compare haptic performance in NAVGPs before and after training on the aforementioned action-based video games.

The current study included only men, because it is hard to find women with the same experience in playing action video games, as has been reported by others (e.g., Green et al. 2012). It would, however, be very interesting to study effects of action video gaming on haptic parallelity matching in women. So far, haptic parallelity studies including both genders have found that men are better at haptically making two bars parallel than women. It has been hypothesized that this is due to the fact that women are less able to overcome the egocentric bias of the hands than men (Van Mier 2014, 2016; Zuidhoek et al. 2007). Only when the influence of the hand was reduced (Van Mier 2013, 2019) or eliminated (Kappers and Schakel 2011; Van Mier 2016), this gender effect disappeared. Additionally, when manipulations favoring more allocentric referencing were introduced, female participants showed improved performance, but to the same extent as male participants, still showing significant gender differences (Zuidhoek et al. 2007; Van Mier 2013, 2020). If women are indeed less able to ignore the bias of the egocentric reference frame, we would expect that female AVGPs would show larger deviations in haptic parallelity matching than male AVGPs, but smaller deviations than female NAVGPs. Replication of this study including females with action video game experience is encouraged.

As mentioned before, we cannot exclude that our results are influenced by the fact that individuals who have better spatial processing or who are more allocentrically oriented might be drawn to playing action video games more fervently, and specifically games with a bird’s-eye perspective. However, several studies have shown that when non-video gamers were trained on action video gaming, their performance on perceptual and cognitive tasks improved even after short periods of training (Feng et al. 2007; Green and Bavelier 2003, 2006, 2007; Green et al. 2012; Li et al. 2009; Spence et al. 2009; Strobach et al. 2012; Wu et al. 2012). The fact that participants who were trained on non-action video games did not show enhanced perceptual and cognitive functioning in the above-mentioned studies strongly suggests a causal relationship between video game playing and perceptual and cognitive enhancement. Lynch et al. (2010) reviewed 12 studies that researched a possible link between video game experience and surgical ability. They reported that there is some evidence that laparoscopic ability improved after short periods of video game practice, suggesting a causal link. However, Boot et al. (2008) did not find enhanced performance on most of the tested cognitive tasks in non-gamers after extensive video game practice. Therefore, the possibility that enhanced performance might be due to self-selection in playing action video games cannot be definitely excluded.

One could argue that the enhanced performance of the AVGPs in our study might be attributed to different expectations regarding their performance in this group. Because we explicitly recruited avid action video gamers, they might have been more motivated to perform well than NAVGPs (Boot et al. 2011; Kristjánsson 2013). However, this might mainly play a role when transfer to more game-like computer tasks is measured. Because the haptic parallelity task is very different from any action video game, we have no reason to assume that AVGPs might have linked their gaming experience to this task. We therefore believe that differences in performance between the groups are not related to differences in expectations and motivation. Furthermore, studies that used covert recruitment reported similar beneficial effects of video gaming as studies in which participants were recruited overtly (Dale and Green 2017b; Donohue et al. 2010; Dye et al. 2009). Additionally, Bediou et al. (2018) did not find evidence for this so-called expectation hypothesis in their meta-analysis regarding the impact of video gaming on cognitive processing.

A limitation of our study is the fact that the control group consisted of NAVGPs who did not play or hardly played action video games. We can therefore not rule out that the observed results are not related to the genre of the action video game played but merely to the fact of playing action video games. It is conceivable that improved performance in the haptic parallelity task would also have been observed in participants playing other action video games, like FPS games, due to the fact that these games enhance visual spatial processing which is transferred to the haptic domain. A study that compared effects of action video gaming in FPS and RTS players found that RTS players had marginally better perceptual sensitivity in a multiple object tracking (MOT) task (Dobrowolski et al. 2015). The authors suggest that this might be due to the fact that RTS games and the MOT task share a common demand with respect to visual attention resources. The “common demands” hypothesis (Oei and Patterson 2014a, b) in the current study would refer to the fact that RTS games and haptic parallelity matching share the common demand of allocentric processing. Future studies should examine if the genre of the action video game differentially affects performance in haptic parallelity matching in AVGPs.

## Conclusion

While previous research has shown that playing action video games modifies visual spatial processing with transfer to new visual contexts, the current findings suggest that action video gaming might also positively benefit haptic spatial processing. We found that AVGPs performed significantly better than NAVGPs in haptic parallelity matching. The results from the current study seem to be consistent with the assumption that playing action video games might also enhance spatial relations in the haptic modality, although a direct causal link still has to be established. These findings add to the accumulating body of evidence supporting the beneficial and versatile effects of playing action video games. It remains to be seen, however, if the current findings can be replicated in a larger sample group, can be observed in non-action video gamers after training, and whether they are specifically related to playing RTS video games or to playing action video games in general.
